# Reprogramming of the cambium regulators during adventitious root development upon wounding of storage tap roots in radish (*Raphanus sativus* L.)

**DOI:** 10.1242/bio.039677

**Published:** 2019-02-20

**Authors:** Ana Cecilia Aliaga Fandino, Hyoujin Kim, Jesse David Rademaker, Ji-Young Lee

**Affiliations:** 1 School of Biological Sciences, College of Natural Science, Seoul National University, 1 Gwanak-ro, Gwanak-gu, Seoul 08826, Republic of Korea; 2 Department of Behavioural Biology, University Utrecht, Padualaan 8, Utrecht 3584CH, The Netherlands; 3 Plant Genomics and Breeding Institute, Seoul National University, 1 Gwanak-ro, Gwanak-gu, Seoul 08826, Republic of Korea

**Keywords:** Radish, Adventitious root, Cambium, Auxin, *WOX11*, *PXY-WOX4*

## Abstract

Cambium contains a stem cell population that produces xylem and phloem tissues in a radial direction during the secondary growth stage. The growth of many storage roots, including in the radish, *Raphanus sativus* L., also depends on cambium. Interestingly, we observed numerous adventitious roots (ARs) emerging from the cambia of cut surfaces when the bases of radish storage tap roots were removed. Previous studies in Arabidopsis showed that the *WOX11/12* pathway regulates AR initiation and meristem establishment in an auxin-dependent manner*.* Here, we provide evidence indicating the evolutionary conservation of the *WOX11/12* pathway during the AR development in radishes. Additionally, we found that expression of two cambium regulators, *PXY* and *WOX4*, is induced in the cambium regions that are connected to emerging ARs via vascularization. Both AR formation and genes associated with this were induced by exogenous auxin. Our research suggests that some key cambium regulators might be reprogrammed to aid in the AR development in concert with the *WOX11/12* pathway.

This article has an associated First Person interview with the first author of the paper.

## Abstract (precis)

**Summary:** Adventitious roots originate from cambia of cut radish taproots. This process depends on the auxin-dependent reprogramming of cambial regulators and the activation of canonical adventitious root regulators.

## INTRODUCTION

The vascular cambium is a meristem organized in radial files between the xylem and the phloem. Cambial initial cells undergo periclinal (asymmetric) division to produce xylem mother cells towards the center of the plant axis and phloem mother cells towards the periphery. Xylem and phloem mother cells divide further and differentiate into cell types constituting secondary xylem and phloem (Miyashima et al., 2013).

In the cambium, signaling sets up the boundary between the xylem and the phloem. Small protein ligands called CLAVATA3/ EMBRYO-SURROUNDING REGION (CLE) 41/44 or TDIF are secreted from the phloem sieve element and diffuse to the cambium where it binds a receptor-like kinase (RLK) called PHLOEM INTERCALATED WITH XYLEM (PXY) or TDR. *PXY/TDR* is specifically expressed in the procambium and cambium (Etchells and Turner, 2010; Fisher and Turner, 2007; Hirakawa et al., 2008). When PXY/TDR interacts with CLE41/44, it triggers two pathways in an independent manner. The first pathway regulates the expression of *WUSCHEL RELATED HOMEOBOX4* (*WOX4*), a gene homologous to *WUSCHEL* (*WUS*), and its redundant gene *WOX14* (Etchells et al., 2013). This plays a big part in the proliferation of vascular stem cells by mediating the auxin responsiveness (Suer et al., 2011). The second pathway is involved in xylem inhibition redundantly with BRASSINOSTEROID-INSENSITIVE 2 (BIN2) (Kondo et al., 2014).

The radish, *Raphanus sativus* L., develops an edible storage taproot. It belongs to the Brassicaceae family, which includes *Arabidopsis thaliana* and species in *Brassica*. Evolutionary proximity of radish to Arabidopsis makes cross-species analyses based on genome information feasible. Its diploid genome is thought to be diverged from the *Brassica* via genome duplication (Mitsui et al., 2015). The growth of radish storage root is driven by high cambium activity in the taproot (Fig. S1A,B). Our previous research has shown that the cell division in the cambium is directly correlated with the girth and yield of storage roots (Jang et al., 2015).

In the presence of wounding or stress, it is a common strategy for plants to repair or regenerate damaged tissues or organs as a survival mechanism. Among many types of plant regeneration, the organogenesis of adventitious roots (ARs) from wounded or detached plant organs has been frequently used as a simple method for vegetative regeneration in agriculture.

Previous research has shown that free auxin accumulates in the wounded organ. Then, high auxin stimulates the transition of a regeneration competent cell to a root founder cell (Hu and Xu, 2016). During this process, auxin activates *WUSCHEL-RELATED HOMEOBOX11* (*WOX11*), a gene that is usually used as root founder cell marker. *WOX11* and its paralog, *WOX12*, activate a set of genes for the development of a root primordia (Liu et al., 2018; Sheng et al., 2017). One of them is *LATERAL ORGAN BOUNDERIES DOMAIN16* (*LBD16*), which functions as a transcriptional regulator involved in the initiation of lateral and adventitious roots (Lee et al., 2017). Additionally, *WOX11* directly activates *WOX5* and *WOX7*, two essential genes for the establishment of a root apical meristem (RAM) (Hu and Xu, 2016).

In this report, we will provide evidence for the radish cambial cells as regeneration competent cells. We will show how the cambium of the taproot reshapes to connect with the AR procambium and reprograms key cambium genes such as *PXY* and *WOX4*, likely for vascularization. Additionally, we will provide support for the evolutionary importance of the *WOX11/12* pathway by describing their expression patterns in developing ARs in radish.

## RESULTS AND DISCUSSION

### Cambium cells are competent for adventitious root formation

Tissues around cambia in cut stems of tomato and *Eucalyptus* have been shown to form AR (de Almeida et al., 2015; Sala et al., 2017). The cambium area has been identified before as an active area during AR and LR formation in woody plants (Chiatante et al., 2010, 2007; de Almeida et al., 2015). In addition, in Arabidopsis the primary roots undergoing the secondary growth, cambium cells could lead to the formation of root founder cells for lateral roots (Baesso et al., 2018). The radish storage taproot rapidly increases its biomass in a radial direction via cambial cell divisions (Jang et al., 2015; Fig. S1A,B). Based on these, we asked whether the cambium tissue serves as a preferential origin of ARs in the radish.

**Fig. 1.**
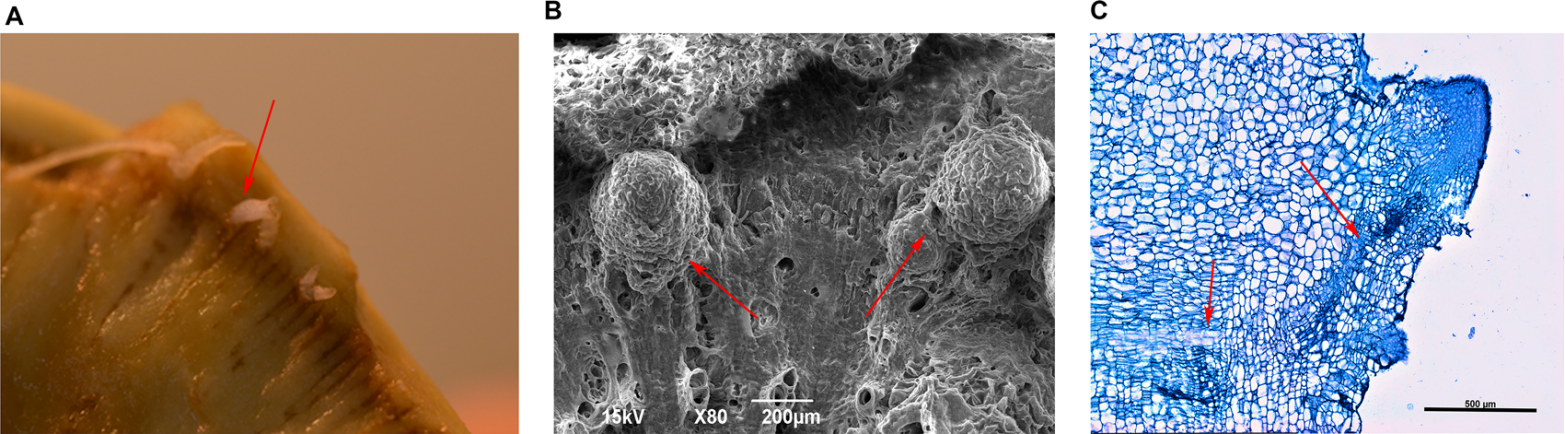
**The vascular cambium is a source of adventitious** **roots.** (A) Image of adventitious roots growing out of the cambium of a taproot of a 7-week-old radish. (B) SEM image of AR primordia coming out of the vascular cambium in 5-week-old radish taproot. (C) Toluidine Blue staining shows cell files (red arrows) connecting between the vascular cambium and the AR primordia. Scale bar: in B, 200 µm; in C, 500 µm.

To induce the AR formation, the base of radish storage taproot was cut off transversely and the remaining root attached to stems and leaves was grown either in soil or in hydroponic media (Fig. S1C). After 2 weeks, we observed the emergence of numerous ARs from cut surfaces. As expected, ARs seemed to appear mostly along the cambium (Fig. 1A). We performed scanning electron microscopy (SEM) and confirmed that AR primordia arose along the cambium (Fig. 1B). Taking an advantage of the feasibility of tracking the cell files in radish taproots, we analyzed how cells are organized in the cambium where ARs emerged (Fig. 1C; Fig. S1C). We noticed the thin layers of cambial cells in the taproot being connected with the AR via strands of small cells (Fig. 1C, indicated by red arrows). These indicated that the cambium in the root undergoing active secondary growth might be reprogrammed to form founder cells of ARs in response to root cutting.

### Auxin responsive WOX11/12 pathway is conserved

**Fig. 2.**
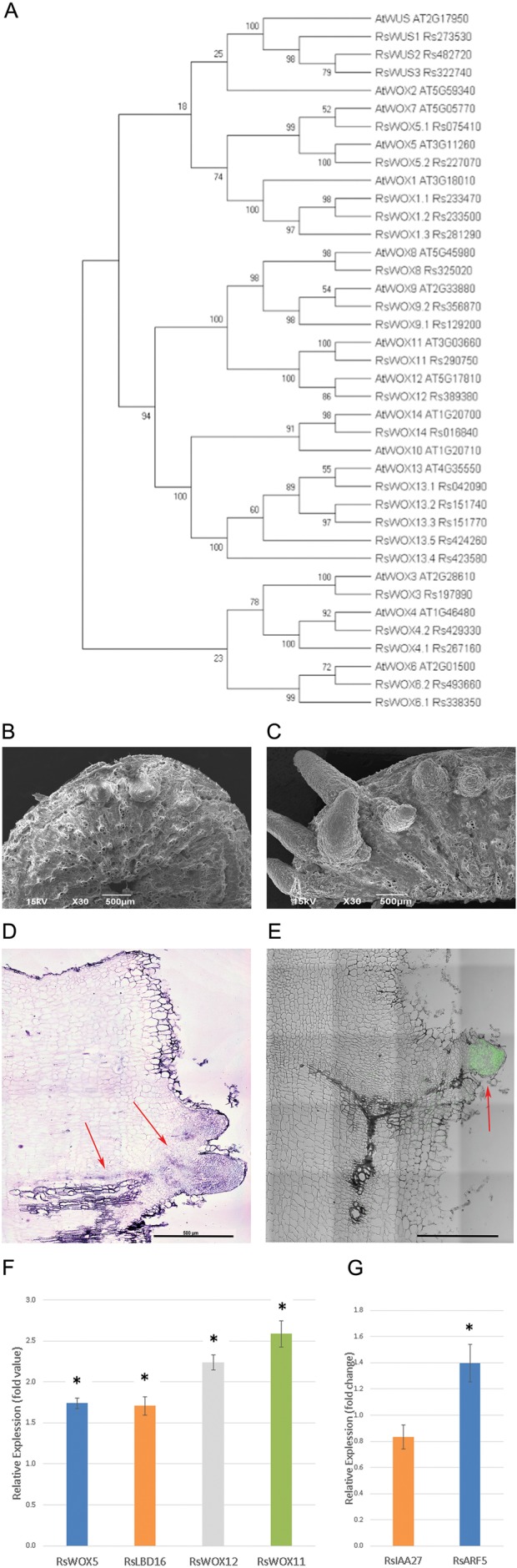
**Detection of *WOX11* pathway and auxin maxima during adventitious root development.** (A) Phylogenetic tree for *WOX* gene family members of *R.* *sativus* and *A.* *thaliana*. (B,C) Scanning electron microscopy of wounded roots incubated in hydroponic media (B) and in hydroponic media supplemented with 1 µM of IAA (C) for 1 week. (D) RNA *in situ* hybridization of antisense probe of *RsWOX11*. Red arrows show the signal in the antisense probe essay. (E) Immunolocalization of PIN1 indicates high auxin distribution in the AR primordium. (F) Relative gene expression of *RsWOX11* pathway genes after 2 weeks of regeneration of 5-week-old radishes in hydroponic media, in comparison to their expression in the root at the beginning of regeneration [fold change=E_sample_^Δct(sample)^ /E_reference_^Δct(reference)^]. (E) Efficiency of a target gene of interest. (G) Relative expression level (fold change) of radish auxin inducible genes *RsIAA27* and *RsARF5* after treatment of 1 µM of IAA in hydroponic media for 1 week. (F,G) Error bar indicates ±s.e., eight biological replicates and three technical replicates. Fold enrichments statistically significant with Student's *t*-test, *P<0.05. Scale bars: 500 µm.

Previous research has shown that the AR formation is regulated by the *WOX11/12* pathway in Arabidopsis and *Populus* (Hu and Xu, 2016; Liu et al., 2014; Sheng et al., 2017). To see whether *WOX11/12* pathway is also associated with the AR formation in radish, we identified the orthologous genes of *WOX11* and *WOX12* in radish and analyzed their expression levels after inducing the regeneration of ARs from cut radish taproots for 2 weeks in hydroponic culture. To find orthologs of *WOX* genes in radish and Arabidopsis, we searched all the WOX homologs in the radish genome database (http://www.radish-genome.org/) and aligned the predicted amino acid sequences using MUSCLE (Edgar, 2004; Fig. S2). We then created a phylogenetic tree using Neighbor-Joining method with bootstrapping 1000 times using MEGA-X (Kumar et al., 2018). *Rs290750* and *Rs389380* were most closely related to Arabidopsis *WOX11* and *12*, thus named as *RsWOX11* and *RsWOX12*, respectively (Fig. 2A). When AR roots were induced, *RsWOX11*, *RsWOX12*, *RsLBD16* and *RsWOX5* were significantly upregulated, supporting evolutionary conservation of *WOX11/12* pathway (Fig. 2F; Fig. S3).

To find the spatial distribution of *WOX11* during AR formation in radish, we performed RNA *in situ* hybridization (Fig. 2D). *RsWOX11* showed expression homogeneous in the AR primordia, which was similar to the expression pattern of *Os-WOX11* in rice during crown root formation (Cheng et al., 2016). This result suggests that the WOX11 pathway is conserved, even between an eudicot and a monocot.

In Arabidopsis AR development, auxin was indicated to play a major role (Sheng et al., 2017). To find whether a similar process operates in radish, we proceeded to regenerate ARs in hydroponic culture for 1 week, by dividing into two groups: one supplemented with 1 µM of auxin (IAA) and the other without auxin (control). For this experiment we used a total of 15 biological replicates for each of the treatments (the control and the auxin group). The experiment was repeated three times. The auxin group had an incidence of AR 1.5 times higher than the control group: an average of 11.13 ARs with a standard error (s.e.) of 2.86 in the auxin group versus 4.53 ARs with a s.e. of 1.93 in the control group. Using Mann–Whitney *U-*test (*P*<0.01), we could verify that the increase of AR formation in the auxin treatment was significant. Representative results are visually shown by scanning electron microscopy (Fig. 2B,C). To check whether this exogenous auxin treatment promoted downstream signaling pathways, we selected and analyzed the expression of *RsIAA27* (Rs048860) and *RsARF5* (Rs414220) and detected the upregulation of *RsARF5* by auxin treatment (Fig. 2G).

These data collectively suggest that auxin promotes the formation of AR primordia. PIN1 is involved in directing auxin flow for vascular formation in developing roots and leaves. We thus analyzed the spatial distribution of PIN1 protein in the cut taproot with emerging AR primordia (Fig. 2E; Fig. S6). As expected, PIN1 proteins were detected throughout the AR primordia as well as the area of vascular connection between the AR and the taproot. This indicates that reprogramming in auxin distribution in the cut taproot might affect the AR formation.

### PXY-WOX4 may be reprogramed to aid the adventitious root formation

**Fig. 3.**
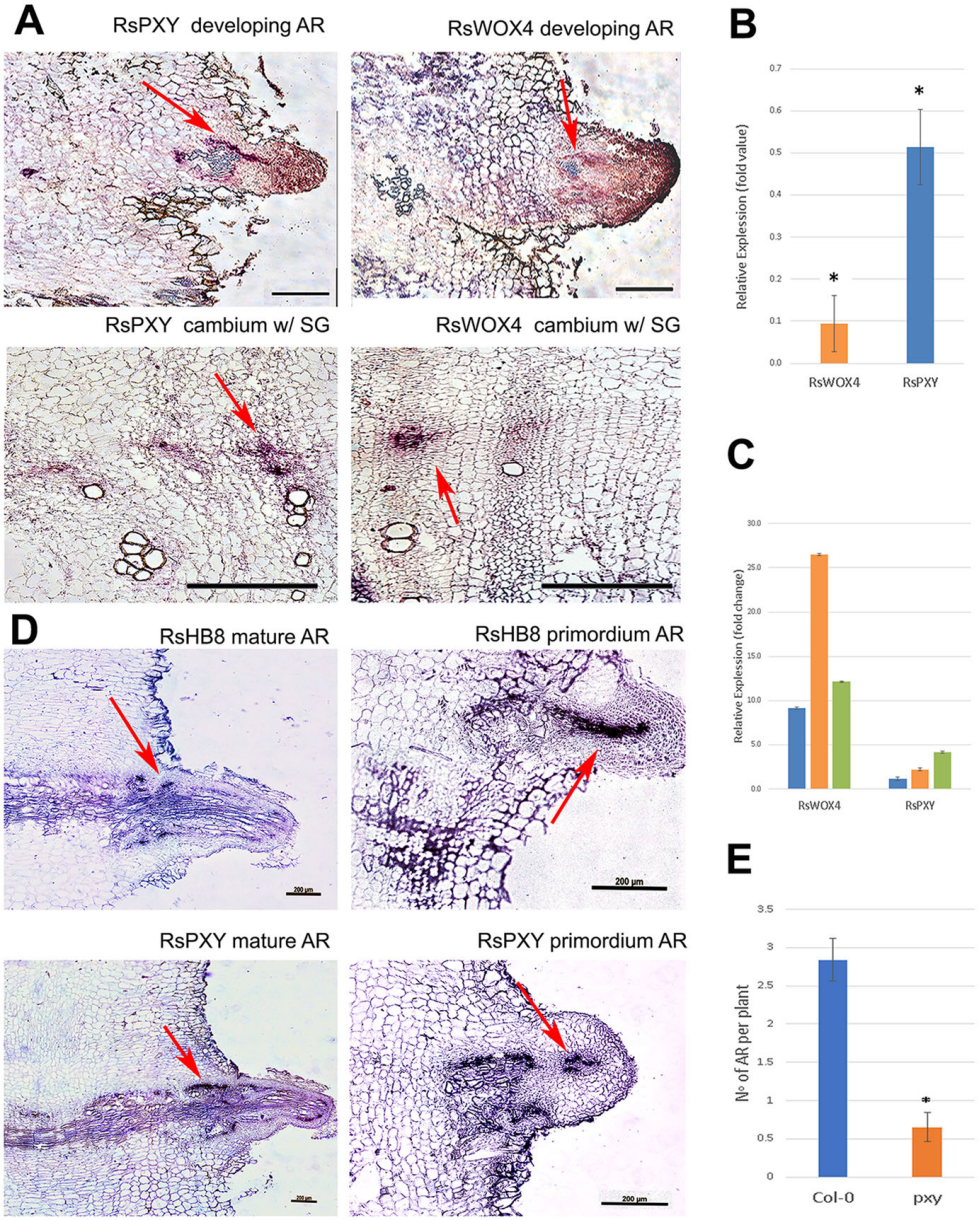
**WOX4 and PXY reprogramming for adventitious root formation.** (A) RNA *in situ* hybridization for antisense *RsWOX4* (right) and *RsPXY* (left) in a developing AR (top) and in a cambium with secondary growth (SG) (bottom). (B,C) Measurement of gene expression changes of *RsWOX4* and *RsPXY* during AR development. (B) The graph shows the relative expression levels of the genes before and after the AR formation. qPCR experiment was performed twice using pooled biological samples with three technical replicates. Error bar indicates ±s.e. (*n*=8). Fold enrichments statistically significant are labeled with asterisks (Student's *t*-test; *P<0.05). (C) The graph shows the relative expression levels of the genes in the AR under auxin treatment in comparison to the non-treated control. qPCR experiment was performed three times using pooled biological samples with three technical replicates. The three bars show the genes’ tendency for upregulation in the biological replicates. Error bar indicates ±s.e. (*n*=3). (D) RNA *in situ* hybridization for antisense *RsPXY* and *RsHB8* in mature AR and AR primordia. (E) The average number of ARs appearing in the wounded area in Col-0 (*n*=23) and *pxy* mutant (*n*=24). Data are presented as mean±s.e. (Mann–Whitney *U-*test; *P<0.01). Scale bars: 200 µm.

Founder cells of ARs seemed to be derived from the cambium. During this reprogramming the cut radish taproots no longer grew in the radial direction as shown in the images of cut radishes pictured after decapitation and after the hydroponic incubation with or without auxin treatment for 7 days (Fig. S4). To find the relationships between changes in cambium activities and their regulators, we analyzed the expression patterns of *RsPXY* and *RsWOX4*. In the intact radish taproot undergoing active secondary growth, these genes showed expression specific to the cambium near emerging xylem vessels (Fig. 3A). When ARs developed from cut roots, we observed the significant reduction of *RsWOX4* and *RsPXY* expression in qRT-PCR, which is consistent with the lack of the cambial activities in the cut roots generating ARs (Fig. 3B). *PXY* and *WOX4* are known to drive the cambium cell proliferation in an auxin-dependent manner (Suer et al., 2011). We observed that auxin promotes AR development from the radish taproot cambium. If *PXY* and *WOX4* are a part of AR development, expression of these genes is likely upregulated upon AR induction in response to auxin treatment (1 µM of IAA). Supporting this idea, when AR formation was induced by incubating cut roots with 1 µM of IAA, expression of both *RsPXY* and *RsWOX4* significantly increased (Student's *t*-test; *P*<0.01) (Fig. 3C). To find how the expression of *RsPXY* and *RsWOX4* was reprogrammed during AR organogenesis, we performed RNA *in situ* hybridization again. *RsPXY* was expressed in a gap between the vascular cambium and the AR primordia, which is where the vascularization of the AR happened (Fig. 3A; Fig. S5). Similarly, *RsWOX4* was present in areas where vascularization was taking place between the AR primordia and the vascular cambium (Fig. 3A; Fig. S5).

Expression of *RsPXY* in a gap between the vascular cambium and the AR primordia was further examined by analyzing its expression in the root generating mature AR. During vascularization, auxin maximum is established along vascular precursors via PIN1. ARF5 activated by auxin then promotes *HB8* transcription and HB8 in turn activates *PIN1* expression. This positive feedback regulation supporting canalization model enables the formation of vascular strands along the auxin flow (Baima et al., 1995, 2001; Scarpella et al., 2006). We thus included *RsHB8* as a vascularization marker and analyzed its expression together with *RsPXY*. *RsHB8* showed an irregular expression pattern with high expression in the areas of vascular initiation during the AR initiation, as expected. Interestingly, *RsPXY* showed the expression pattern very similar to *RsHB8* (Fig. 3D). In the mature AR, *RsPXY* and *RsHB8* were expressed along the AR procambium which connects with the vascular cambium of the cut taproot (Santuari et al., 2011). These expression changes indicate that *PXY*-*WOX4* might function for the AR development when the secondary growth no longer happens in the cut taproot.

Our investigation in radish suggested the recruitment of *PXY* and *WOX4* to the AR development. To find whether these regulators actually affect AR formation from the cut taproots, we counted the ARs in Arabidopsis *pxy* mutant. Col-0 (*n*=23) and *pxy* (*n*=24) plants were grown for 18 days in MS media, their primary roots were decapitated, and then the shoots without roots were grown in B5 media for 4 days (Fig. S7). The number of ARs from cut root surfaces decreased significantly from an average of 2.91 to 0.62 in the *pxy* (Fig. 3E).

In Arabidopsis, WOX11/12 promotes AR founder cells and subsequently activates *LBD16* and *WOX5*, which coordinate the initiation of a root primordium and a root apical meristem (Hu and Xu, 2016). Our expression analyses indicate a similar program might operate in AR development from radish taproots and the cambium serve as foci for the AR initiation (Fig. 1). During AR development, we found the vascular connection being established between the cambium of a cut taproot and the AR primordium.

**Fig. 4.**
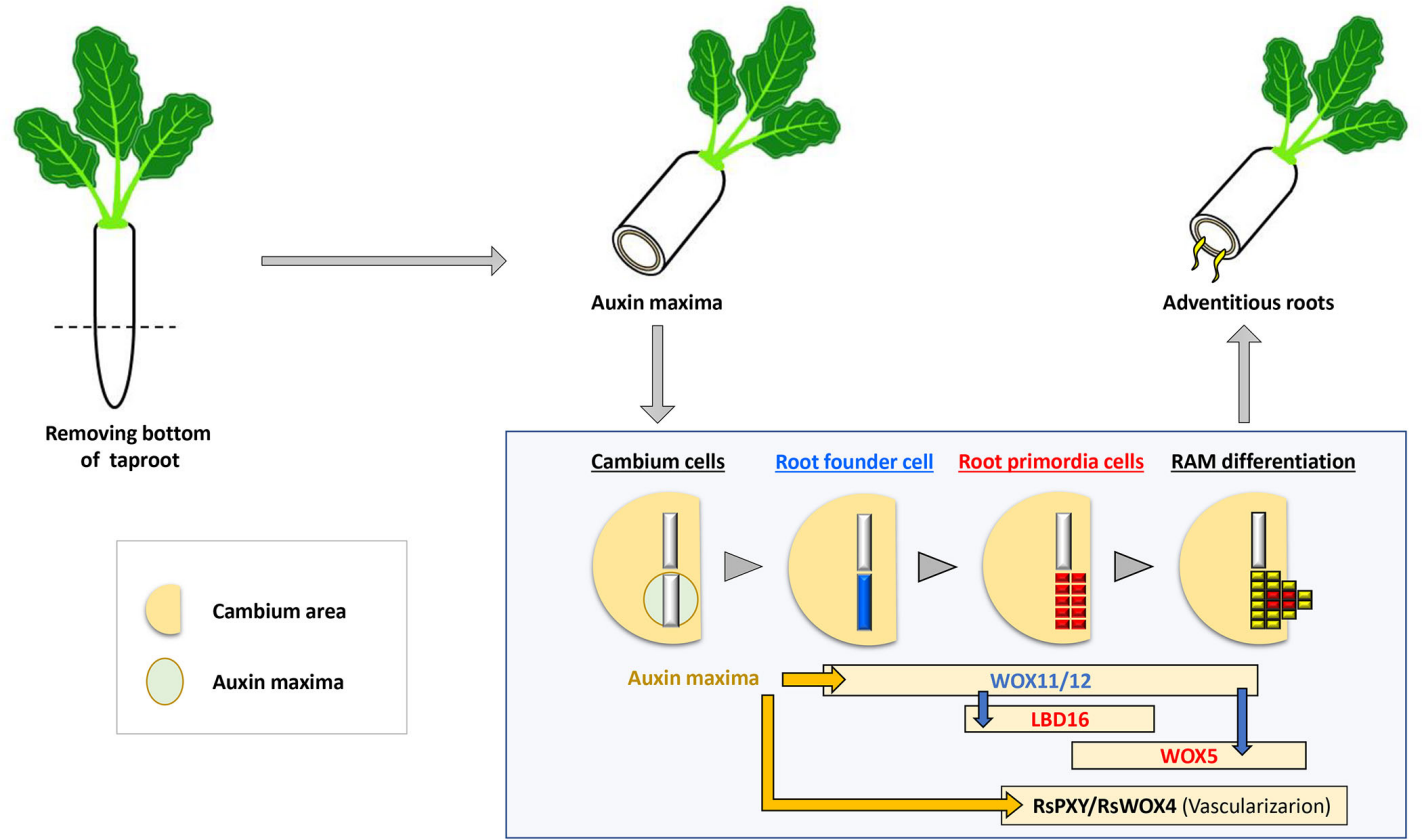
**Model for adventitious root development in radish.** When the bottom part of the taproot is removed, localized auxin maxima are created. In this point, high levels of auxin activate the AR development program: *RsWOX11/12* initiates the formation of AR founder cells from the cambium, and activates *RsLBD16* that initiates the root primordium and *RsWOX5* that establishes the new root apical meristem. Auxin also reprograms the cambium specific *RsPXY-RsWOX4* pathway to induce vascularization in the areas for AR formation, thereby connecting the vascular tissue of the AR to the tap root.

Auxin flow plays a key role in the formation of vascular connections during organogenesis, and *HB8* and *PIN1* promotes this process as parts of positive feedback regulation (Baima et al., 2001; Donner et al., 2010; Scarpella et al., 2006). Consistently, in radish we detected the expression of PIN1 and *RsHB8* in the region where the vascular connection was made between cut taproot cambium and the AR primordium. Exogenous application of auxin to the cut taproot promoted the AR formation specifically from the cambium (Fig. 2). These collectively indicate that the stem cells in the cambium have a capacity to initiate AR formation in an auxin dependent manner. Our further investigation suggests that *RsPXY* and *RsWOX4* that are known to be in charge of cambial cell division during secondary growth (Etchells et al., 2013; Suer et al., 2011) and regulation of xylem differentiation (Fisher and Turner, 2007; Kondo et al., 2014) are reprogramed to re-establish their expression in the junction between AR primordia and the cut taproot. In an RNA *in situ* hybridization, we observed *RsPXY* expression largely disappeared when the secondary growth of a taproot stopped upon the removal of the root base and then re-emerged intermittently where vascularization happened as AR primordia developed. At the point of AR growth, it showed homogeneous expression in the AR procambium that was connected to the vascular cambium (Fig. 3). *RsHB8* also showed expression pattern similar to *RsPXY*, confirming the procambium-cambium connection (Baima et al., 1995; Santuari et al., 2011). Recruitment of *RsPXY* and *RsWOX4* to AR development is also supported by their response to auxin. Exogenous auxin application to the cut taproot no longer promoted the secondary growth, however strongly induced the expression of *RsPXY* and *RsWOX4* (Fig. 4). This process seems functionally important since *pxy* mutant showed significant reduction in the emergence of ARs from the cut taproots in Arabidopsis. We interpret this behavior as a plants' survival strategy that allows them to initiate organ regeneration upon wounding in a time- and cost-efficient way. Unraveling molecular mechanisms underlying how auxin redirects the vascular stem cell function from organ growth to organ regeneration will advance our understanding of the remarkably dynamic nature of plant development.

## MATERIALS AND METHODS

### Plant materials and growth conditions

Radishes for this experiment were grown in a growth room with a constant temperature of 22°C and a photoperiod of 16 h of light and 8 h of darkness. For hydroponic culture, we used Hoagland solution (50% v/v) after adjusting its pH to 7.

### Tissue staining and imaging

Paraplast^®^-embedded tissue sections were prepared on glass slides following the same procedures for the tissue preparation for RNA *in situ* hybridization. For the Toluidine Blue staining, slides were placed in a slide rack and passed through a hydration series of Histoclear^®^ for 10 min two times, 100% ethanol for 2 min two times, 90% ethanol for 2 min, 70% ethanol for 2 min, 50% ethanol for 2 min, and water for 2 min. Then, the tissues on the glass slides were dipped in Toluidine Blue solution (0.001 g in 200 ml) for 20 s and washed in distilled water three times. Finally, slides were processed in a reverse order of a hydration series for mounting. Images were taken with a Nikon eclipse Ni light microscope.

### Real-time quantitative PCR

cDNAs were synthesized and diluted twofold, by mixing 20 µl of Milli-Q water and 20 µl of cDNA. qPCR reactions were set up with 10 µl per reaction as follows: 5 µl of iQ™ SYBR Green^®^ supermix (Bio-Rad, Cat No. 1708880), 0.5 µl forward primer and 0.5 µl reverse primer (10pM), 3 µl of Milli-Q water, and 1 µl of cDNA. Cycling conditions were as follows: 3 min at 95°C, 48 cycles of denaturation for 10 s at 95°C, annealing for 30 s at 56°C, and extension for 30 s at 72°C. qPCR reactions and fluorescence detection were performed using a CFX96 Real-Time PCR machine (Bio-Rad). RsActin was used as a reference gene in order to assess gene expression levels of our genes of interest. To perform the qPCR reactions, specific primers (Table S3) were designed using Primer3 (Rozen and Skaletsky, 2000).

### Scanning electron microscopy

Radish storage tap roots grown for 5 weeks post seed planting were cut transversely and upper parts of plants were grown in hydroponic media for 1 week. Root segments with emerging ARs from cut surface were fixed overnight at 4°C in 25% of Glutaraldehyde in 25 mM of phosphate buffer (pH 7). The next day, the fixative was poured off and 1% of osmium tetroxide solution was added. The vials were incubated at 4°C for 2 days. The samples were thoroughly rinsed in 25 mM of phosphate buffer (pH 7) and then dehydrated in an ethanol series. Specimens kept in 100% of ethanol were critical-point dried, coated with gold particles and then imaged under a scanning electron microscope (JSM-6390LV, JEOL).

### RNA *in situ* hybridization

#### cDNA library preparation

For preparing the cDNA library, total RNA from 7-week-old radish roots was isolated using the QIAGEN RNeasy^®^ Plant Mini kit (Qiagen Cat No./ID: 74903). For the cDNA library preparation, a reverse-transcription reaction was carried out using 1μg of the total RNA with the Invitrogen™ SuperScriptIII reverse transcriptase kit (Thermo Fisher Scientific, Cat No.180800930) following the manufacturer's instruction.

#### Gene cloning

To clone our selected genes, gene-specific forward and reverse primers were designed and obtained (Table S1). Genes were PCR-amplified from the cDNA library obtained in the previous step. The product was run through an electrophoresis on 1% agarose gel and purified by the QIAquick^®^ Gel Extraction Kit (Qiagen, Cat No. 28704). The purified product was inserted into pENTER/D-TOPO vector with the pENTER™/D-TOPO^®^ Cloning Kit Invitrogen™ (Thermo Fisher Scientific, Cat No. K240020). Gene clones were verified by Sanger sequencing in NICEM at Seoul National University.

#### RNA probe preparation

Template DNAs for RNA probes were synthesized by PCR amplification of a part of genes cloned in the previous section. Primers used can be found in Table S2. After gel purification, the DNA template concentration should be at least 100 ng/µl. The transcription using T7 RNA polymerase was carried with the DIG labeling Kit (Sigma-Aldrich, Cat No. 11175025910). *In vitro* transcription mixture was prepared by adding 2 µl of acetylated BSA, 2 µl of transcription buffer, 2 µl of 10% Triton X-100, 2 µl of 10XNTP labeling mixture and 1 µl of RNase inhibitor with a final volume of 9 µl. To this mixture, 9 µl of purified PCR product and 2 µl of T7 RNA polymerase were added. The reaction was incubated at 37°C for 3 h and then DNA was removed from the mixture by adding 5 µl of DNase buffer, 22 µl of RNase Free water and 2 µl of RNase-free DNase. The mixture was incubated for 15 min. at 37°C. Finally, the probe was let to precipitate overnight at −80°C by adding 6 µl of 4 M LiCl, 1 µl of 0.5 M EDTA and 180 µl of 100% ethanol. The next day, RNA was precipitated by centrifugation at 13,000 rpm for 30 min. at 4°C. RNA pellet was washed with ethanol, dried, and dissolved in RNase-free water.

#### Tissue preparation

The radish root segments with AR regeneration were collected and fixed in 4% paraformaldehyde. The tissue was vacuum-infiltrated for 5 min three times, and incubated overnight at 4°C in fresh fixative. The next day, the fixative was removed and the tissue was rinsed four times with 1X Phosphate-Buffered Saline (PBS) for 15 min each time.

Tissue was then dehydrated using the following ethanol series (25%, 50%, 75%, and three times in 100% of ethanol), and then infiltrated with a series of Histoclear^®^ diluted in 100% of ethanol: 25%, 50%, 75%, and three times of 100% Histoclear^®^. Finally, a half of the tube was filled with Paraplast^®^ chips and left overnight at 58°C for infiltration. For the following 4 days the Paraplast^®^ was replaced twice a day and then the tissue was imbedded with Paraplast^®^ in a mold and let solidify. Tissue blocks were sliced using a RM 2255 microtome (Leica) in 15 µm of thickness and then mounted on a TRUBOND^®^ (Thermo Fisher Scientific, Cat No. NC0270688).

#### Slide pretreatment

Slides were placed in a rack and passed through the following solution series in RNase free conditions. To begin with, slides were placed two times in Histoclear^®^ for 10 min. Then the slides were passed through ethanol series (100%, 100%, 95%, 85%, 70%, 50%, 30% of ethanol diluted in 0.85% of NaCl) for 1 min in each solution. Afterwards slides were placed in a series of following solutions: 0.85% of NaCl for 2 min, 0.2 M of HCl for 20 min, RNase-free water for 5 min, 1×PBS for 2 min. Then, the tissue sections on slides were treated with Pronase enzyme (0.135 mg/ml; Sigma-Aldrich, Cat. No.P6911) for 28 min at 37°C. Reaction was stopped with 0.2% of glycine in 1×PBS, washed in 1×PBS for 2 min, and fixed in 4% of paraformaldehyde for 10 min. Slides were rinsed for 2 min in 1×PBS, then submerged in 0.85% of NaCl for 2 min and finally treated in an ethanol series in a reverse order of dehydration. Slides were dried in RNase free conditions for 1 h.

#### Prehybridization and hybridization

Slides were placed in the container that soaked with 50% of formamide. Each slide with tissue sections was coated with 250 µl of prehybridization solution (50% formamide, 1× salts, 1× Denhardt's, 200 µg/ml of tRNA, 10 U/ml of RNase inhibitor). Slides were incubated for 1 h at room temperature and then 1 h at 45°C. Meanwhile, RNA probes were denatured by incubating at 80°C for 1 min and moving into ice. After the prehybridization, each slide was treated with 250 µl of the hybridization solution (50% formamide, 1.25×salts, 12.55% dextran sulfate, 250 µg/ml tRNA, 1.25 Denhardt's, 12.5 U/ml of RNase inhibitor, 1.5 µg/ml/kb of probe, DEPC water) and incubated in the 50% formamide chamber at 45°C for 24 h.

#### Post-hybridization washes

After the hybridization, slides were the placed in a rack which stands in a jar with 0.2× of Saline Sodium Citrate Buffer (SSC) for 1 h at 55°C. The solution was later replaced and incubated for 1 h. After finishing the washes, slides were rinsed in NTE solution (10 mM of Tris pH8.0; 5 mM EDTA) and incubated for 30 min at 37°C in a solution of 10 µg/ml RNase A in 0.5 M NaCl; NTE. Slides were rinsed for 5 min in NTE and incubated for 1 h in 0.2XSSC at 55°C. Finally, slides were rinsed in 1×PBS.

#### Signal detection

Slides were placed in Blocking solution [100 mM Tris, 100 mM NaCl, 1% blocking reagent of DIG Nucleic Acid detection kit (Roche Cat No. 11175041910)] with gentle agitation for 45 min. Then the blocking buffer was replaced by buffer A (100 mM Tris, 100 mM NaCl, 1%BSA, 0.3% Triton X-100) and slides were incubated for another 45 min. Antibody conjugate from the DIG Nucleic Acid detection kit was spread on the slides in a 1:1000 ration in Buffer A. Slides were incubated for 2 h with high humidity. After this step, slides were washed in Buffer A three times for 20 min each time and then twice in Detection Buffer (100 mM Tris pH9.5, 100 mM NaCl, 50 mM MgCl2) for 5 min each time. Finally, slides were incubated with 500 µl of color substrate (200 µl of NBT/BCIP solution DIG Nucleic Acid detection kit in 10 ml of detection buffer and 100 µl of levamisole) at room temperature for 24 h in the dark condition.

### Immunolocalization with PIN1 antibody

Paraplast-embedded tissue sections were prepared on glass slides following the same procedure as the tissue preparation for RNA *in situ* hybridization. For dewaxing, glass slides were incubated in Histoclear^®^ for 15 min at room temperature, transferred to 100% ethanol for 10 min two times, and then air-dried for 15 min. Then, slides were treated in a following rehydration series: fresh 100% ethanol, 90% of ethanol diluted in ddH_2_O, two times with 70% ethanol diluted in 1×PBS, 50% and 25% of ethanol diluted in 1×PBS, and two times with 1×PBS. In each step, slides were incubated for 10 min at room temperature. 150 µl of blocking solution [2% BSA fraction V (Sigma-Aldrich A-3912) in 1×PBS] was spread on each slide and incubated in a humid chamber for 30 min at room temperature. After washing glass slides in 1×PBS, we applied 500 times diluted PIN1 antibody (Cat#:R2114-2, Rabbit polyclonal, Abiocode) in a blocking solution. After incubating overnight at 4°C, glass slides were washed in 1×PBS six times, for 10 min at room temperature each time. We applied glass slides with 200-fold diluted secondary antibody [Alexa Fluor^®^ 488 F(ab’) 2 fragment of goat anti-rabbit IgG, IgM (H+L), Invitrogen™] in a blocking solution. We incubated the glass slides in a humid chamber in the dark for 1 h at room temperature. And then glass slides were washed six times in 1×PBS, for 10 min at room temperature each time. The glass slides were mounted with ddH_2_O and signals were detected and imaged using Laser Scanning Confocal Microscope (Leica SP8) with the excitation/emission wavelength of 488 nm/505 to 530 nm.

## Supplementary Material

Supplementary information
