# Supplementary material for: Reprogramming of the cambium regulators during adventitious root development upon wounding of storage tap roots in radish (Raphanus sativus L.)
Source: Biol Open. 2019 Feb 20;8(3):bio039677. doi: 10.1242/bio.039677 (PMC6451342; doi:10.1242/bio.039677)
Supplement: Supplementary information [file biolopen-8-039677-s1.pdf]

Supplementary Information

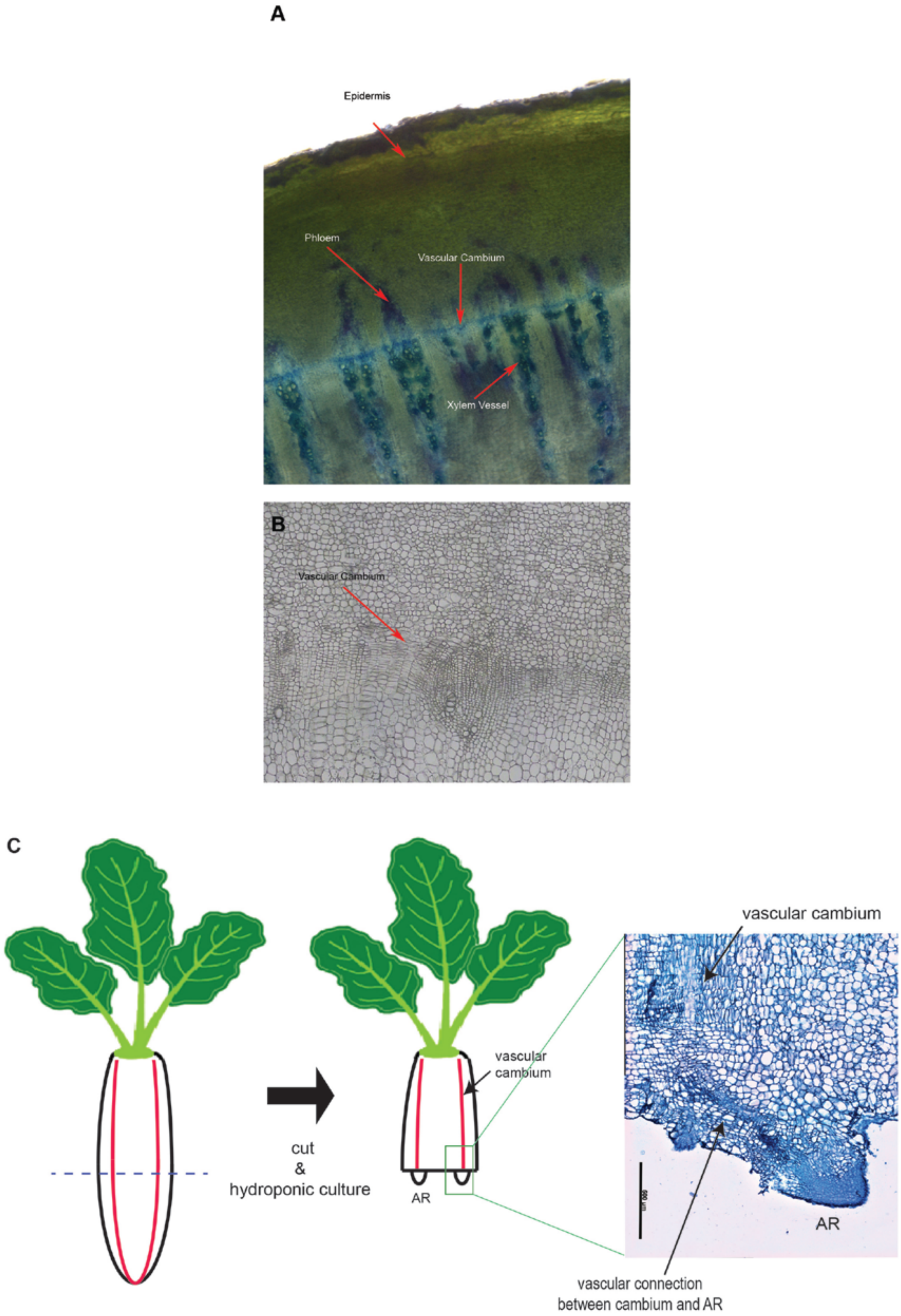

Figure S1. Tissue organization of radish tap root and a schematic of adventitious root emergence from a cut radish tap root. (A) Hand cross section of 5-week-old radish tap root

Biology Open • Supplementary information

stained in toluidine blue. Pointed tissues show distinctive staining patterns. Magnification 40X. (B) Close-up view of panel (A) using thin paraffin sectioning. It shows cellular organization around vascular cambium. Thin-layered cells pointed in a red arrow constitute vascular cambium. (C) Schematic of analysis AR anatomy from a cut radish tap root shown in Fig. 1C and images for RNA *in situ* hybridizations.

Biology Open • Supplementary information

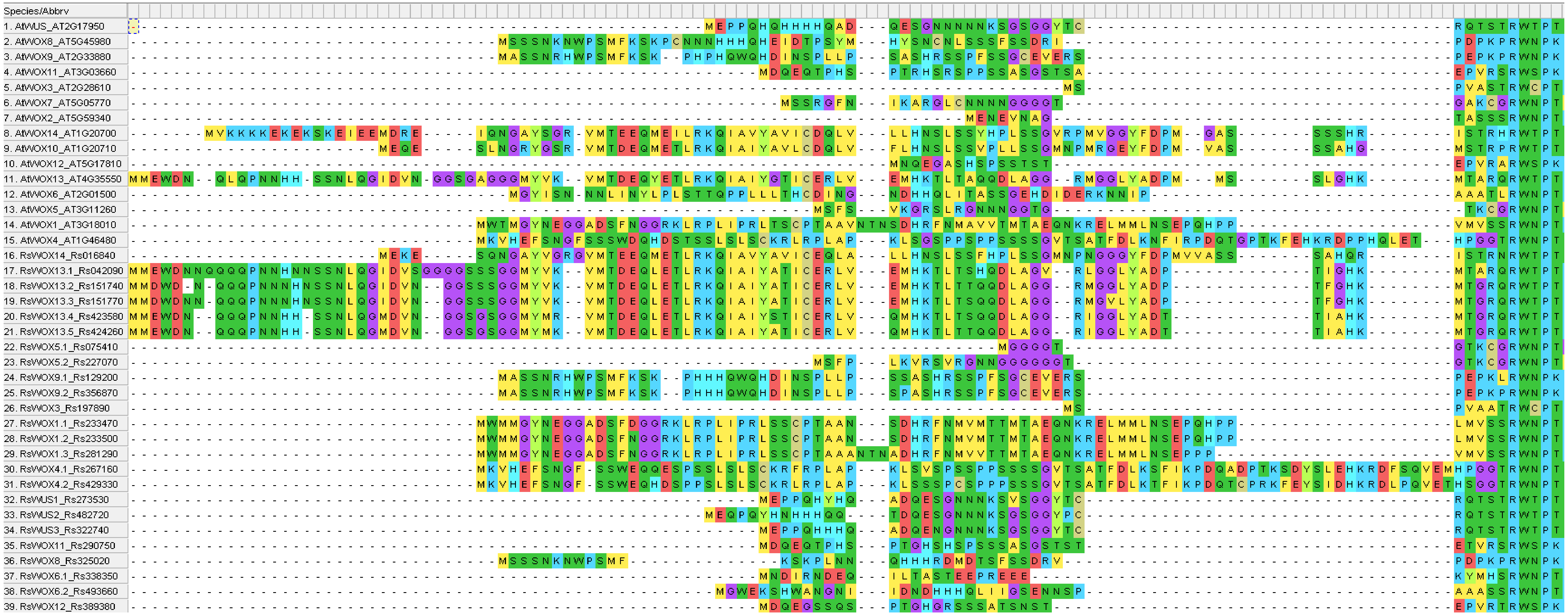

Biology Open • Supplementary information

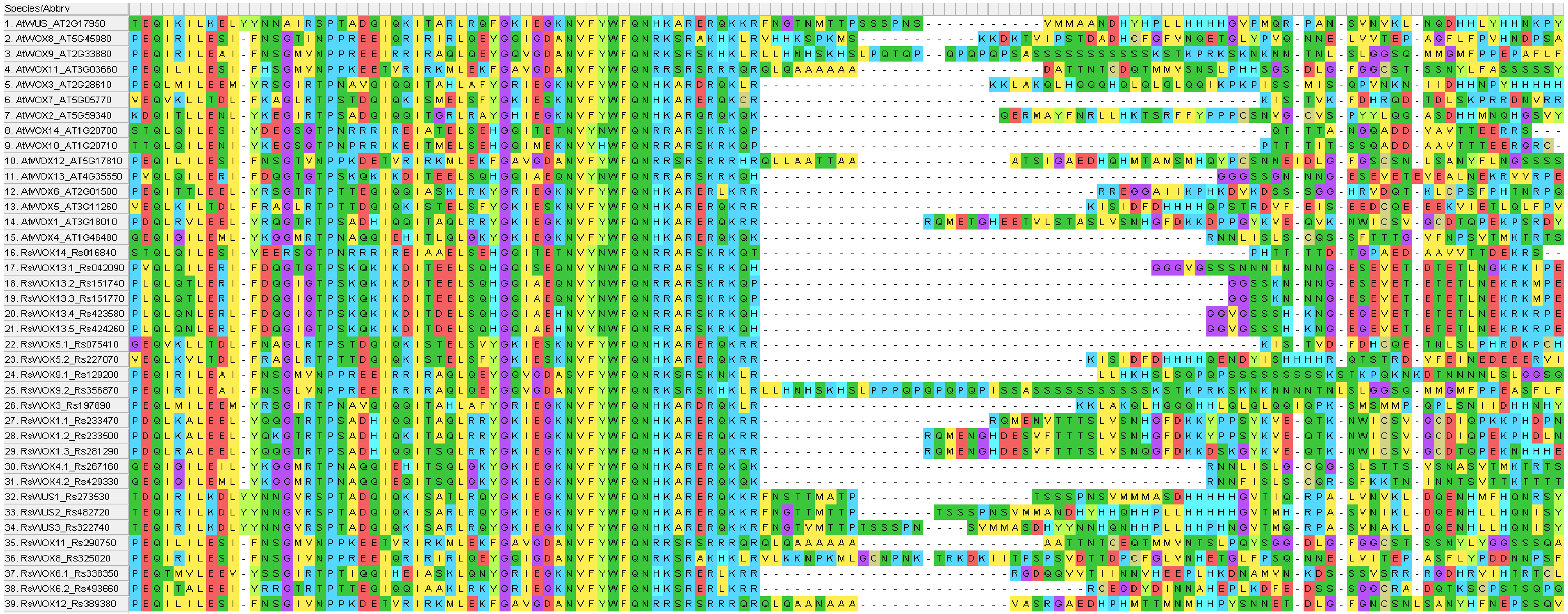
Species/Abbrv
1. AtWUS_AT2G17950
2. AtWOX8_AT5G45980
3. AtWOX9_AT2G33880
4. AtWOX11_AT3G03660
5. AtWOX3_AT2G28610
6. AtWOX7_AT5G05770
7. AtWOX2_AT5G59340
8. AtWOX14_AT1G20700
9. AtWOX10_AT1G20710
10. AtWOX12_AT5G17810
11. AtWOX13_AT4G35550
12. AtWOX6_AT2G01500
13. AtWOX5_AT3G11260
14. AtWOX1_AT3G18010
15. AtWOX4_AT1G46480
16. RsWOX14_Rs016840
17. RsWOX13.1_Rs042090
18. RsWOX13.2_Rs151740
19. RsWOX13.3_Rs151770
20. RsWOX13.4_Rs423580
21. RsWOX13.5_Rs424260
22. RsWOX5.1_Rs075410
23. RsWOX5.2_Rs227070
24. RsWOX9.1_Rs129200
25. RsWOX9.2_Rs356870
26. RsWOX3_Rs197890
27. RsWOX1.1_Rs233470
28. RsWOX1.2_Rs233500
29. RsWOX1.3_Rs281290
30. RsWOX4.1_Rs267160
31. RsWOX4.2_Rs429330
32. RsWUS1_Rs273530
33. RsWUS2_Rs482720
34. RsWUS3_Rs322740
35. RsWOX11_Rs290750
36. RsWOX8_Rs325020
37. RsWOX6.1_Rs338350
38. RsWOX6.2_Rs493660
39. RsWOX12_Rs389380

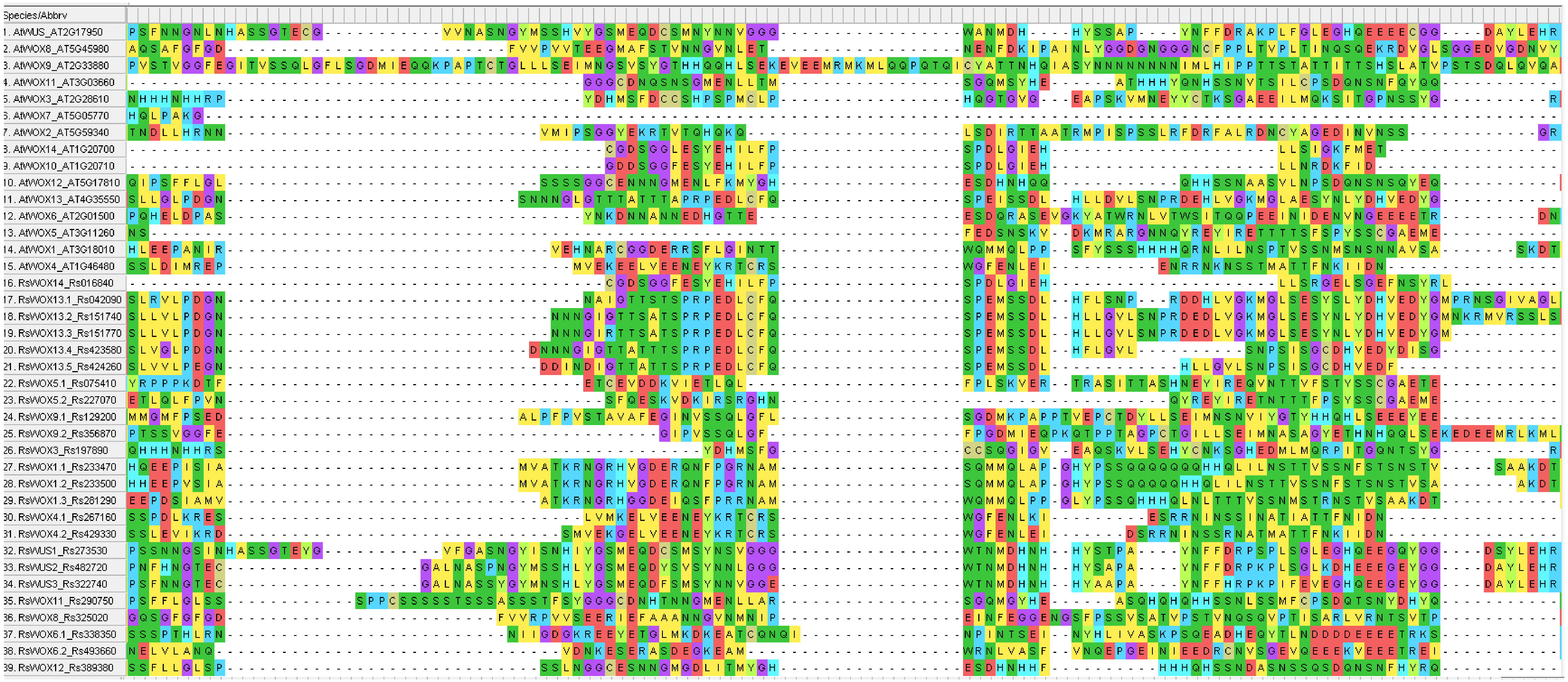
Species/Abbrv
1. AtWUS_AT2G17950
2. AtWOX8_AT5G45980
3. AtWOX9_AT2G33880
4. AtWOX11_AT3G03660
5. AtWOX3_AT2G28610
6. AtWOX7_AT5G05770
7. AtWOX2_AT5G59340
8. AtWOX14_AT1G20700
9. AtWOX10_AT1G20710
10. AtWOX12_AT5G17810
11. AtWOX13_AT4G35550
12. AtWOX6_AT2G01500
13. AtWOX5_AT3G11260
14. AtWOX1_AT3G18010
15. AtWOX4_AT1G46480
16. RsWOX14_Rs016840
17. RsWOX13.1_Rs042090
18. RsWOX13.2_Rs151740
19. RsWOX13.3_Rs151770
20. RsWOX13.4_Rs423580
21. RsWOX13.5_Rs424260
22. RsWOX5.1_Rs075410
23. RsWOX5.2_Rs227070
24. RsWOX9.1_Rs129200
25. RsWOX9.2_Rs356870
26. RsWOX3_Rs197890
27. RsWOX1.1_Rs233470
28. RsWOX1.2_Rs233500
29. RsWOX1.3_Rs281290
30. RsWOX4.1_Rs267160
31. RsWOX4.2_Rs429330
32. RsWUS1_Rs273530
33. RsWUS2_Rs482720
34. RsWUS3_Rs322740
35. RsWOX11_Rs290750
36. RsWOX8_Rs325020
37. RsWOX6.1_Rs338350
38. RsWOX6.2_Rs493660
39. RsWOX12_Rs389380

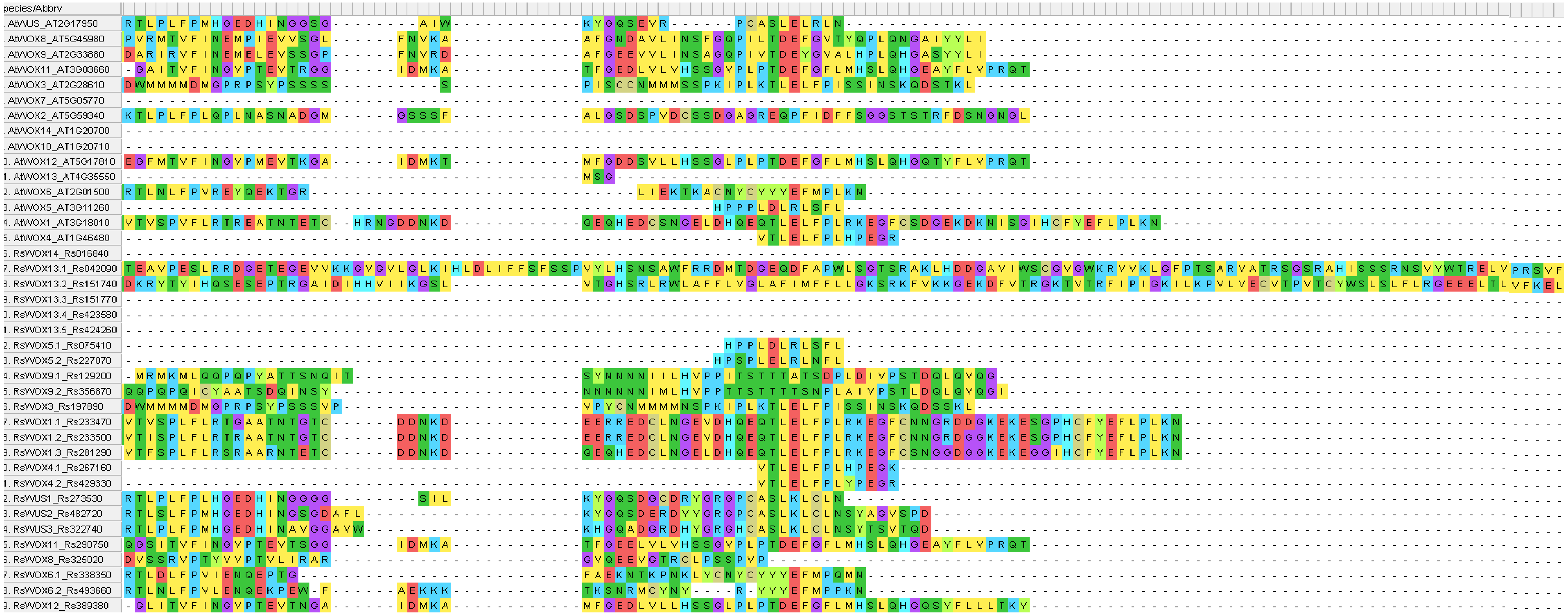
pecies/Abbrv
. AtWUS_AT2G17950
. AtWOX8_AT5G45980
. AtWOX9_AT2G33880
. AtWOX11_AT3G03660
. AtWOX3_AT2G28610
. AtWOX7_AT5G05770
. AtWOX2_AT5G59340
. AtWOX14_AT1G20700
. AtWOX10_AT1G20710
0. AtWOX12_AT5G17810
1. AtWOX13_AT4G35550
2. AtWOX6_AT2G01500
3. AtWOX5_AT3G11260
4. AtWOX1_AT3G18010
5. AtWOX4_AT1G46480
6. RsWOX14_Rs016840
7. RsWOX13.1_Rs042090
8. RsWOX13.2_Rs151740
9. RsWOX13.3_Rs151770
0. RsWOX13.4_Rs423580
1. RsWOX13.5_Rs424260
2. RsWOX5.1_Rs075410
3. RsWOX5.2_Rs227070
4. RsWOX9.1_Rs129200
5. RsWOX9.2_Rs356870
6. RsWOX3_Rs197890
7. RsWOX1.1_Rs233470
8. RsWOX1.2_Rs233500
9. RsWOX1.3_Rs281290
0. RsWOX4.1_Rs267160
1. RsWOX4.2_Rs429330
2. RsWUS1_Rs273530
3. RsWUS2_Rs482720
4. RsWUS3_Rs322740
5. RsWOX11_Rs290750
6. RsWOX8_Rs325020
7. RsWOX6.1_Rs338350
8. RsWOX6.2_Rs493660
9. RsWOX12_Rs389380

Biology Open (2019): doi:10.1242/bio.039677: Supplementary information

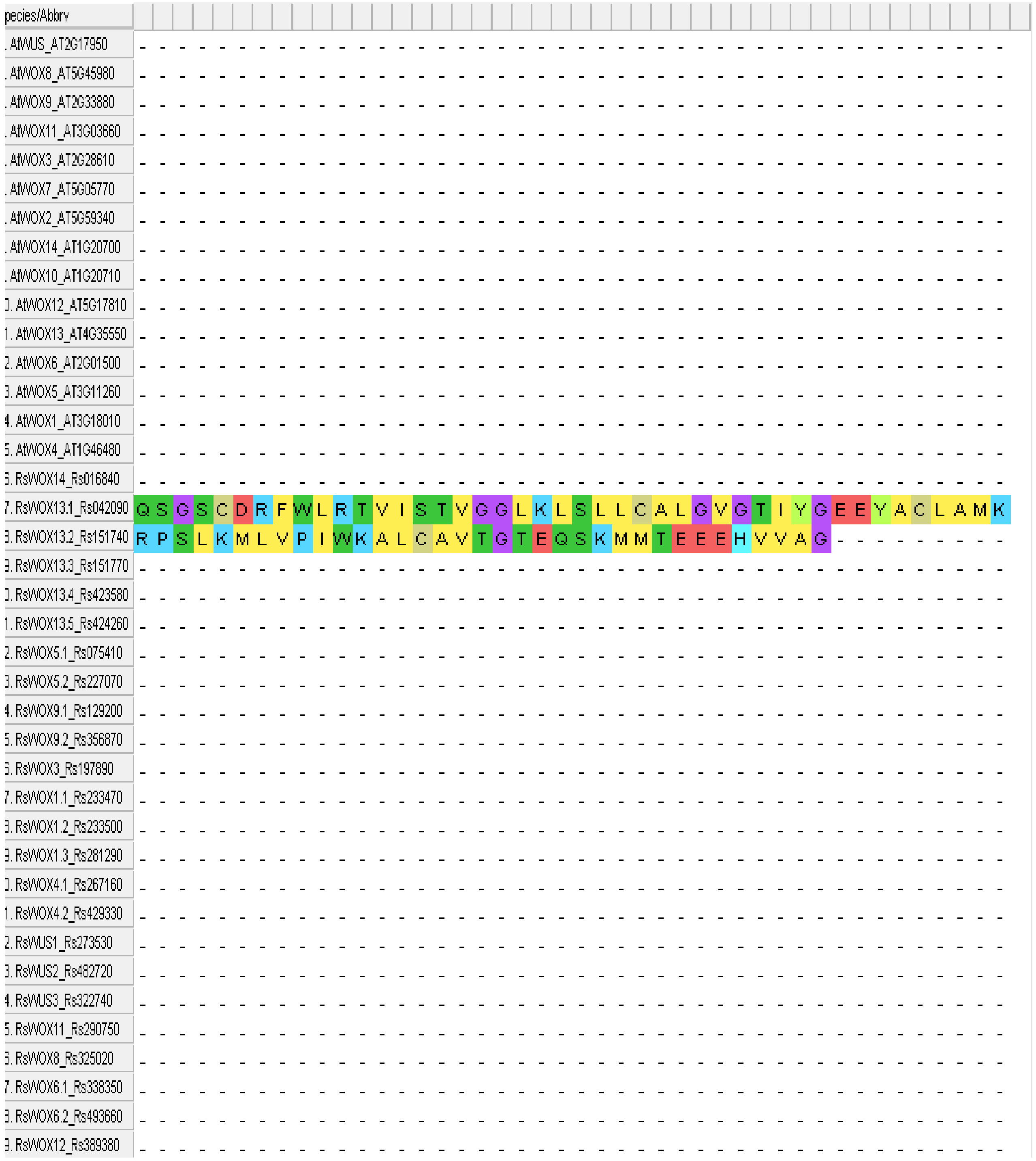

Figure S2. Amino acid sequence alignment of WOX gene family members in *Raphanus sativus* and *Arabidopsis thaliana* using MUSCLE.

Biology Open • Supplementary information

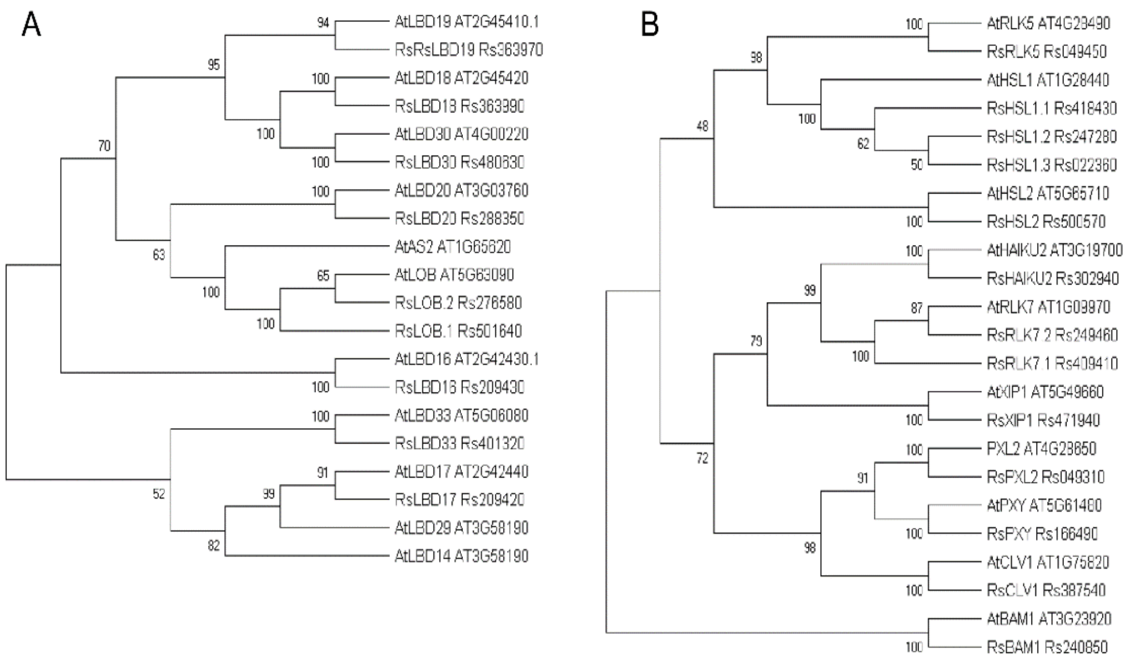

Figure S3. (A) Phylogenetic tree for *LBD* gene family group of *Raphanus sativus* and *Arabidopsis thaliana*. (B) Phylogenetic tree for *RLK* gene family group of *Raphanus sativus* and *Arabidopsis thaliana*. The tree was constructed using the Neighbor-Joining method. The bootstrap consensus tree was inferred after 1000 times of replications. The evolutionary distances were computed using the JTT matrix-based method. Evolutionary analyses were conducted in MEGA X.

Biology Open • Supplementary information

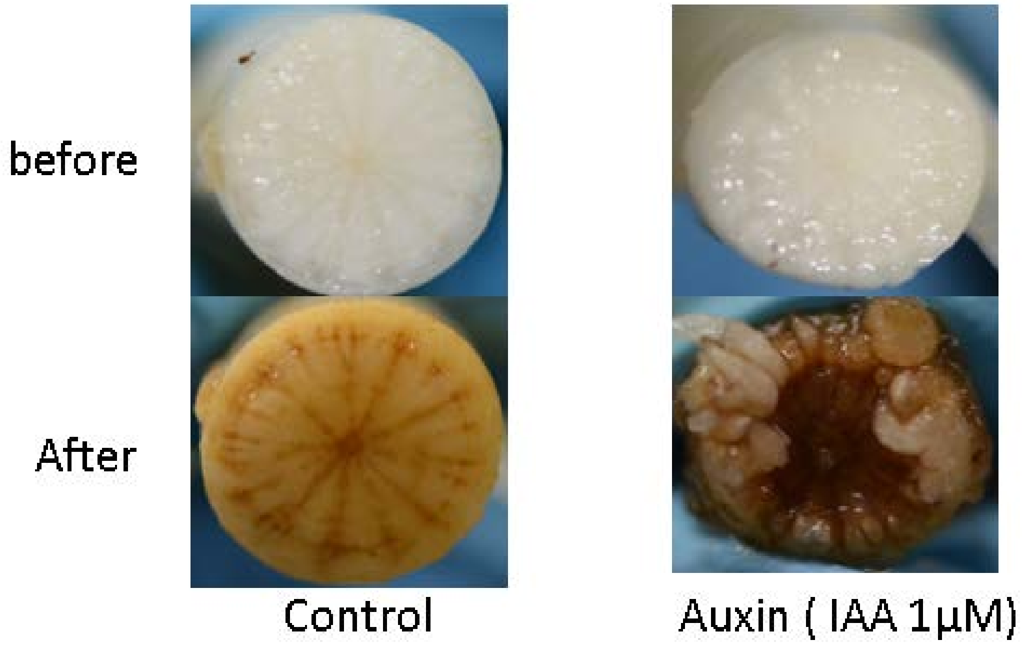

Figure S4. Images of wounded roots right after the cut and 1 week later with (Auxin) and without (control) 1 µM of IAA.

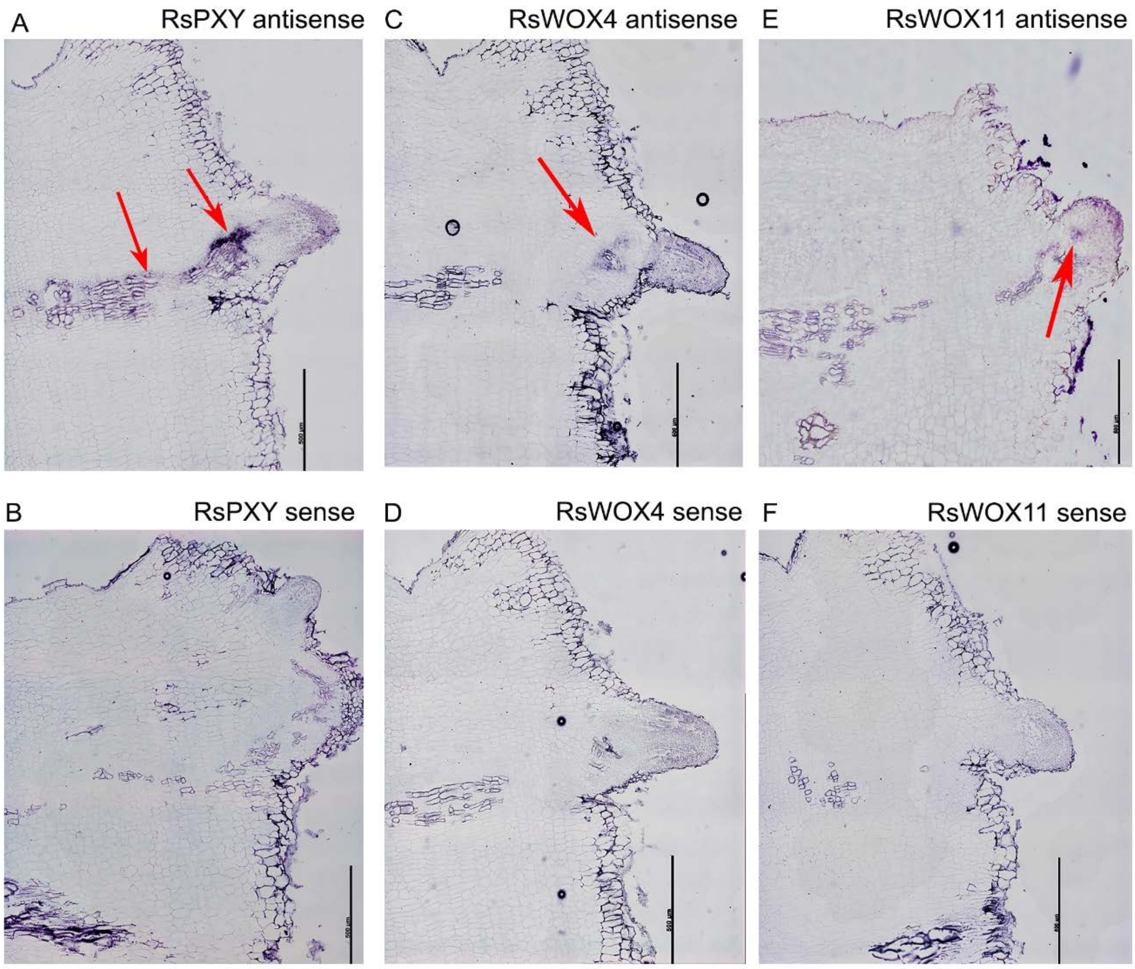

Figure S5. RNA *in situ* hybridization of *RsPXY* , *RsWOX4* and *RsWOX11* on emerging AR from root cuts. 5-week-old radish storage tap roots were cut transversely and ARs were regenerated for 2 weeks by incubating the shoot side of roots in the hydroponic culture system. (A) Antisense *RsPXY* probe. (B) Sense *RsPXY* probe. (C) Antisense *RsWOX4*. (D) Sense *RsWOX4* (E) Antisense *RsWOX11* (F) Sense *RsWOX11*. Red arrows indicate where RNA expression is detected. Scale bars 500µm.

Biology Open • Supplementary information

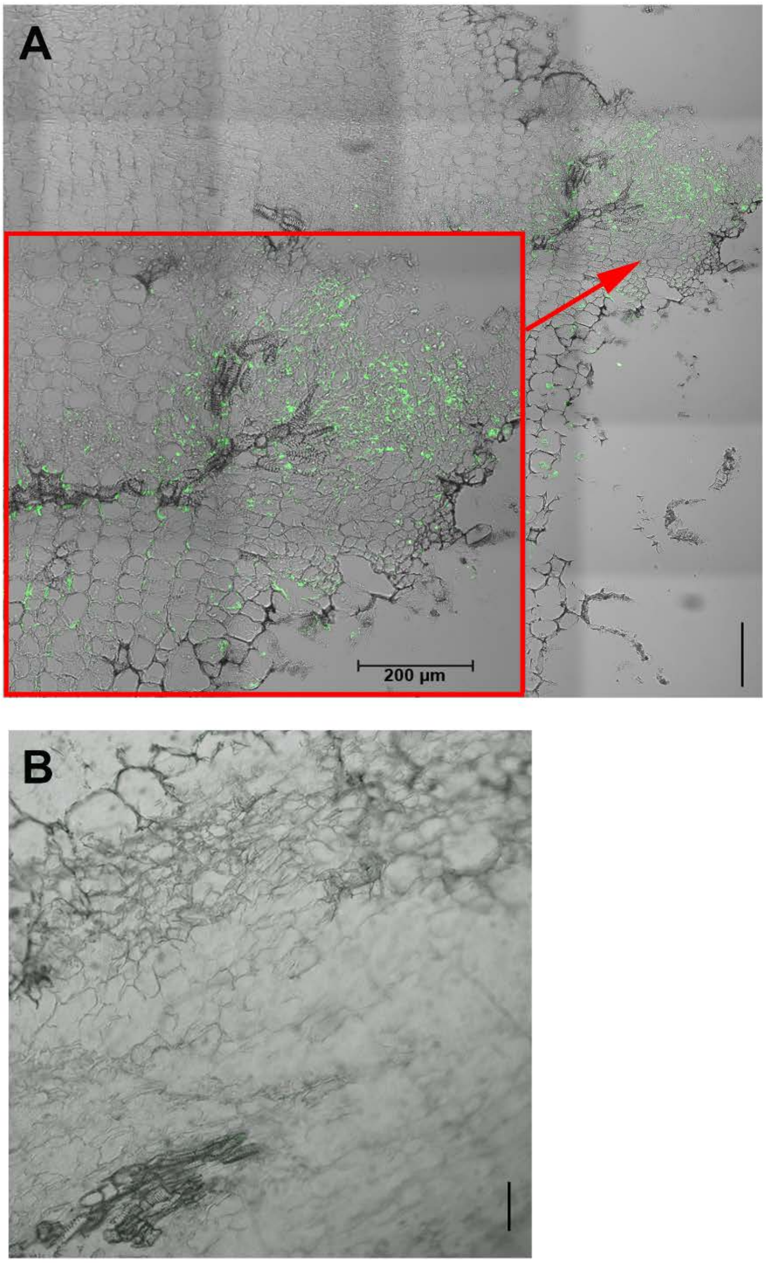

Figure S6. Immunolocalization of PIN1. (A) Signals are detected along the vascular connection between the main root and the AR. (B) Negative control that was not treated with PIN1 antibody. Scale bars, 200µm.

Biology Open • Supplementary information

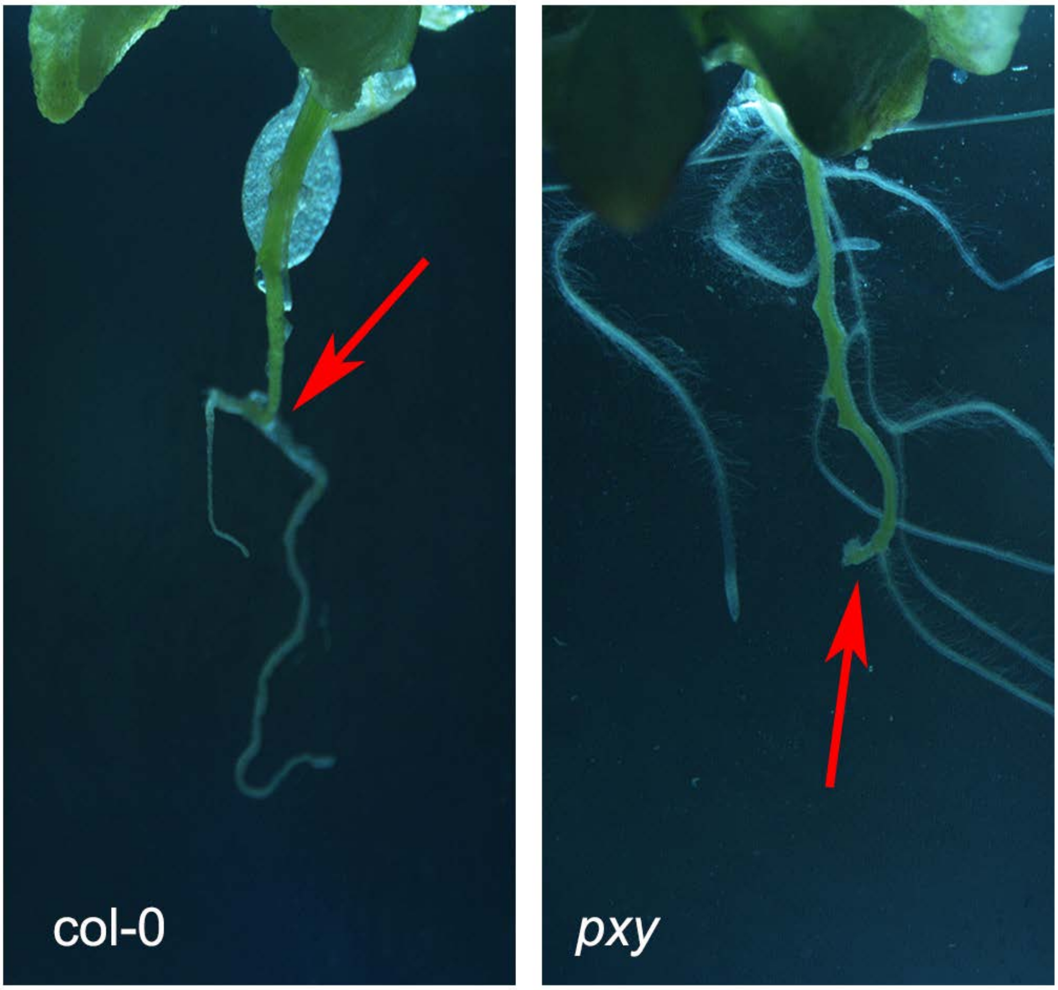

Figure S7. Regeneration of ARs. Primary roots of 18-day old Arabidopsis Col-0 (left) and *pxy* (right) were cut and the emergence of ARs were imaged after regeneration in B5 media for 4 days.

Biology Open • Supplementary information

Table S1. Primers used for cloning.

Oligo Name	Oligo Seq (5’-> 3’)
RsWOX11_F	CACCATGGACCAAGAACAAACACCAC
RsWOX11_R	TGTCTGTCTTGGAACCAGGAA
RsHB8_F	CACCATGGGAGGAGGGAGCAATAG
RsHB8_R	AATAAAAGACCAGTTGAGGAACATG
RsWOX4_F	CACCATGAAGGTTCATGAGTTTTCCAATC
RsWOX4_R	TTTACCTTCAGGGTGCAAAGG
RsPXY_F	CACCATGAACAAGAAGAACACTTCTCTTTCTCTTG
RsPXY_R	TCACACTCCAATCTTCTTCTGACATTTAACAC

Biology Open • Supplementary information

Table S2. Primers used for making the RNA in-situ probes.

Oligo Name	Oligo Seq (5'-> 3')
RsWOX11_sense_F	GGATCCTAATACGACTCCTATAGGGAGGATGGACC AAGAACAAACACCAC
RsWOX11_sense_R	GAAAAAGTTGAAGAGCTAGCTG
RsWOX11_antisense_R	GGATCCTAATACGACTCACTATAGGGAGGGAAAAA GTTGAAGAGCTAGCTG AAG
RsHB8_sense_F	GGATCCTAATACGACTCACTATAGGGAGGATGGGA GGAGGGAGCAATAGTA
RsHB8_sense_R	CTGCAATTCCCGTGCATCCA
RsHB8_antisense_R	GGATCCTAATACGACTCACTATAGGGAGGCTGCAA TTCCCGTGCATCCA
RsHB8_sense _F2	GGATCCTAATACGACTCACTATAGGGAGGTGCAAA TGTGCAG TGGAGT
RsHB8_sense_ R2	TACATTCTCTTGCATGTGCATCTC
RsHB8_antisense_F2	TGCAAATGTGCAGTGGAGT
RsHB8_antisense_R2	GGATCCTAATACGACTCACTATAGGGAGGTACATT CTCTTGCATG TGCATCTC
RsWOX4_sense_F	GGATCCTAATACGACTCACTATAGGGAGGGAAGCA GAAGAGGAACAACCT A
RsWOX4_sense_R	TGCAAAGGAAAAAGCTCAAGGG
RsWOX4_antisense_F	GAAGCAGAAGAGGAACAACCTCA
RsWOX4_antisense_R	GGATCCTAATACGACTCACTATAGGGAGGTGCAAA GGAAAAAGCTCAAGGG
PXY_sense_F	GGATCCTAATACGACTCACTATAGGGAGGGCTTTC ACCTCAACTCTCCTCC
RsPXY_sense_R	TCCAAAGGCAGCAGCAAGCCTTCGAA
RsPXY_antisense_F	GCTTTCACCTCAACTCTCCTCC
RsPXY_antisense_R	GGATCCTAATACGACTCACTATAGGGAGGTCCAAA GGCAGCAGCAAGCCTTCGAA

Biology Open • Supplementary information

Table S3. Primers used for quantitative RT-PCR

Oligo Name	Oligo Seq (5'-> 3')
RsWOX5 (Fw)	CGACGGTGGAGCAGTTGA
RsWOX5 (Rv)	CGGCGTTTTTGCCTCTCTCT
RsWOX11 (Fw)	TGGTGGGTCATCATCTCAAGC
RsWOX11 (Rv)	CCCATTTGACCAGACCTTGC
RsWOX12 (Fw)	TCGTCACAAAGCCCTACTGG
RsWOX12 (Rv)	TTAGGTGACCAACGTGTCCG
RsPXY (Fw)	GCGACAATGCAACTTCTCAA
RsPXY (Rv)	GCTGATGTCGAGAGTGGTGA
RsHB8 (Fw)	ATGGGAGCCTTGTGATATGC
RsHB8 (Rv)	CCATGGCTCAAGATCGAAAT
RsLBD16 (Fw)	TTGCTCAATGTTCCAATCCA
RsLBD16 (Rv)	TTCATTTGCAGGACTTGTGC
RsActin (Fw)	GCATCACACTTTCTACAAC
RsActin (Rv)	CCTGGATAGCAACATACAT
RsWOX4 (Fw)	AGCCGATCCAACAAAATCTG
RsWOX4(Rv)	ATCTGTTGCGCGTTAGGAGT
RsIAA27(Fv)	AGTTGGGTTCATGTCGCTCA
RsIAA27(Rv)	GGAACGGCCGGTTTAATGTC
RsARF5(Fw)	TCACCTCAGGACTCAAACGC
RsARF5(Rv)	AGCTGCTCTGAAGCCATGTT

Biology Open • Supplementary information
